# From discovery to application: merging modern omics with traditional hypothesis-driven approaches in muscle myopathy studies

**DOI:** 10.3389/fphys.2024.1520196

**Published:** 2025-01-20

**Authors:** Wei Guo, Sandra G. Velleman, Gale M. Strasburg

**Affiliations:** ^1^ Department of Animal and Dairy Sciences, University of Wisconsin-Madison, Madison, WI, United States; ^2^ Department of Animal Sciences, The Ohio State University, Wooster, OH, United States; ^3^ Department of Food Science and Human Nutrition, Michigan State University, East Lansing, MI, United States

**Keywords:** hypothesis-testing approach, hypothesis-generating approach, omics, meat quality, muscle myopathy

## Introduction–A historical perspective

The conventional wisdom within the scientific community dictates that a complete understanding of the factors that determine the quality of muscle as a food requires that we define the molecular mechanisms of muscle growth and development, of the function of muscle as an organ, and ultimately of *postmortem* conversion of muscle tissue to meat. As a corollary, understanding the mechanistic underpinnings that lead to myopathies such as wooden breast, white striping, or pale, soft, exudative meat would–ideally–be applied by breeders, producers, and processors to prevent or mitigate meat quality problems. However, the growing gap between knowledge accumulation and its application suggests that we pause and reconsider our approach to addressing scientific questions.

Interestingly, the gap between knowledge accumulation and application has deep roots in our field. In 1965 and 1969, the University of Wisconsin-Madison hosted two international, ground-breaking symposia on “The Physiology and Biochemistry of Muscle as a Food.” Attendees of this interdisciplinary conference included medical scientists, physiologists, biochemists, pathologists, nutritional scientists, and meat scientists, all sharing the goal of better understanding the relationship between the biology of living muscle and the quality of muscle tissue as a food.

In the first chapter of the proceedings of the second meeting (Marsh et al., 1970), published in 1970, Dr. B. Bruce Marsh quoted from a paper by Prof. E. C. Bate-Smith published in 1948 in the first Volume of *Advances in Food Research*:

“In the past two decades … fundamental knowledge of the physiological and biochemical properties and behavior of muscle has increased out of all recognition. Perhaps because of the bewildering rate of growth of this fundamental knowledge and the constantly changing conception of muscle which has resulted, there has not been … any striking application of the principles of modern biochemistry to the technology of handling of meat animals and meat.”

Marsh goes on to observe:

“… one's attention is drawn … to [Bate-Smith’s] thoughts on the bewildering growth of fundamental knowledge, on ‘the constantly changing conception of muscle,’ and on the absence of ‘any striking application of these principles’ to meat technology. These views are even more relevant today than they were in 1948; for although our knowledge of meat and its qualities has grown enormously in the intervening years, knowledge of muscle and its behavior has increased even more. The gap between discovery and application is still widening. The output of new information, the development of new concepts, the postulation of new theories --- these are accelerating at such a rate that we may liken muscle biology to an expanding universe and ourselves to the particles within it, fast receding from each other and suffering the inevitable consequence of greater and greater isolation. Compounding the problem, new branches of science splinter from the old, and studies which a few years ago were clearly together within one particular discipline are now so altered that they are scarcely recognizable as relatives.”

Since those words were published over five decades ago, we have witnessed an exponential growth of these “new branches of science” and a shift in our approach to doing science. In the following sections, we contrast the historical approach to addressing scientific questions with that which is increasingly dominant in today’s culture. We then illustrate with examples the importance of using hypothesis-generating (data-driven) science to generate hypotheses that address mechanistic questions. Finally, we propose a framework for integrating the two approaches and application of knowledge.

## Present-day challenges: hypothesis-based vs. hypothesis-generating research

Historically, science made incremental advances by asking a question based on observations, formulating a hypothesis, explaining the observations, and testing that hypothesis with an appropriate experimental design. The results of the experiment(s) would be analyzed and interpreted, and the conclusions drawn would lead to a new hypothesis.

Contrast this hypothesis-based approach with that used today by many muscle biologists (including ourselves) as we routinely employ techniques from these “new branches of science” such as nucleic acid sequencing, transcriptomics, proteomics, lipidomics, metabolomics to address broad-based biological questions. For example, how do the muscles of the meat animal respond to heat or cold stress, and what are the implications for meat quality?

Applying transcriptome analysis, for example, may provide information on genes that are up- or downregulated in response to a biological perturbation or stress. These genes may be organized into clusters that are associated with specific biological pathways and functions, and in turn, may suggest a hypothesis to be tested that would not only define a mechanism by which muscle responds to the stress, but also, in so doing, may suggest a dietary or other management intervention that may result in reduced incidence of myopathy due to a stress, and improvement in meat quality.

We contend that collectively, we all too frequently conduct and publish our studies that conclude with pious words such as “these results provide new information that may be useful for breeders and producers for improvement of avian health and meat quality.” If that is our endpoint, we have missed the opportunity to generate hypotheses that could lead to discovery of mechanisms that indeed lead to strategies that can be applied at the level of breeder and producer.

This shift in research paradigms introduces both opportunities and challenges. On one hand, hypothesis-generating research allows the discovery of new mechanisms that may otherwise remain uncovered. On the other hand, it can lead to data overload, making it difficult to pinpoint the most significant findings. To fully utilize the power of -omics research, scientists must integrate these exploratory methods with traditional hypothesis-driven research. Although exploratory data alone may provide new insights, it is critical that researchers develop testable hypotheses from these findings, ensuring that molecular discoveries lead to practical, mechanistic insights. By integrating these approaches, we can narrow the gap between data collection and actionable applications. In the following section, we offer examples of work that illustrate this approach.

## Examples of -omics research as hypothesis-generating research

Example 1: Sporer et al. (2011) investigated temporal changes in breast muscle gene expression in two lines of turkeys at three stages of muscle development: 18d embryo (when the breast muscle is undergoing hyperplasia), 1-day post-hatch (when muscle is undergoing hypertrophy), and 16 weeks (market age of the turkey). Of the >3,000 differentially expressed genes (FDR <0.0001), three were selected for further investigation for their role in muscle growth and development: versican (*VCAN*), matrix Gla protein (*MGP*), and death-associated protein (*DAP*). These selections were based on the magnitude of expression changes with developmental stage coupled with lack of information on their roles in myogenesis (Velleman et al., 2012). Velleman et al. then used small interfering RNA (siRNA) in cultured satellite cells to characterize the roles of these genes in proliferation and differentiation (Velleman et al., 2012). Subsequent studies further documented the critical role of DAP in satellite cell proliferation and differentiation (Shin et al., 2013; Horton et al., 2020). Although the significance of these genes is now evident, the next steps should extend this work to define the specific functions of these gene products.

Example 2: Lake et al. (2021) used genome-wide association and transcriptome studies to identify genes associated with the wooden breast myopathy in broilers. Velleman and colleagues then selected the top differentially expressed genes in Wooden Breast that are not typically associated with the growth or regeneration of skeletal muscle for further study in a cell culture system examining their effect on satellite cell activity (Velleman et al., 2022; Velleman et al., 2024). Of the genes studied, Calponin 1 (CNN1) and PHD and RING finger domain-containing protein 1 (PHRF1) were the most promising targets, as they were the top genes affected by Wooden Breast determined by Genome Wide Association studies. The follow-up studies on these genes examined these novel genes for their effects on satellite cells. CNN1 is a smooth muscle protein that binds F-actin and tropomyosin. In Wooden Breast myopathy-affected muscle, sarcomere integrity is lost, and CNN 1 may be involved in the maintenance of actin filament integrity. Like CNN1, PHRF1 was not previously reported in skeletal muscle until the study by Lake and colleagues (Lake et al., 2021). PHRF1 stabilizes genomic integrity with DNA damage and in tumorigenesis (Chang et al., 2015). Wooden Breast results in DNA damage; PHRF1 may localize in Wooden-Breast-affected muscle to areas of genomic damage and be involved in the stabilization of these lesions. Like Example 1, the next steps should target the specific mechanisms by which these proteins function.

## A call for deeper mechanistic understanding

A balanced research approach that integrates both strategies would be ideal for translating molecular discoveries into meaningful biological insights and practical applications. One of the greatest challenges researchers face when transitioning from hypothesis-generating to hypothesis-driven research is selecting the right target gene(s) from the vast -omics dataset. Although most researchers focus on the greatest -fold changes (highest or lowest), this approach can lead to false starts. Genes that show dramatic changes in expression may not be the most important contributors to the observed phenotype, while some key regulatory genes or non-coding RNAs, which act as “fine-tuners,” may exhibit only modest expression changes. The overwhelming amount of data generated by -omics research often makes it difficult to discern which gene(s) are truly driving a particular phenotype.

To overcome this issue, a more systematic and integrative approach to gene selection is needed. We offer the following thoughts as a possible strategy for breeders to consider in the selection of genes for further investigation:

1. Network and Pathway Analysis: Rather than focusing solely on the highest or lowest expressed genes, integrating systems biology approaches—such as gene regulatory network or pathway analysis—can help prioritize genes that play central roles in biological processes. By understanding how genes interact within networks, researchers can identify key nodes that may have outsized influence on phenotype, even if their expression levels are not extreme.
2. Functional Annotation and Validation: Combining -omics data with existing knowledge of gene function can guide researchers toward more promising candidates. Databases that annotate gene function, evolutionary conservation, or known involvement in specific biological processes can provide context for selecting target genes. Furthermore, targeted functional studies (e.g., RNAi, CRISPR/Cas9) can quickly validate the role of key genes.
3. Integration of Multiple Layers: Cross-referencing data from multiple -omics layers (e.g., transcriptomics, proteomics, and metabolomics) can improve gene selection accuracy. For example, if a gene shows differential expression at the RNA level and corresponding changes at the protein or metabolite level, this strengthens the case for its functional relevance.
4. Phenotype-Gene Correlation Models: Developing computational models that link gene expression patterns to specific phenotypic traits can aid in the identification of causal genes. Machine learning techniques, in particular, can be used to predict which genes are most likely responsible for observed phenotypes, helping to narrow down targets for experimental validation.

Due to space limitations of an opinion paper, a comprehensive discussion of strategies to extend experimental results to applications for use by breeders and producers is beyond the scope of this article. However, researchers may refer to previous studies that have proposed detailed strategies aimed at improving traits and achieving desired phenotypes, such as enhanced meat quality characteristics (Te Pas et al., 2017; Rexroad et al., 2019).

## Conclusion

In the 54 years since Marsh noted the widening gap between discovery and application, this separation has exponentially intensified. Despite the remarkable advances in our understanding of muscle biology, driven largely by the explosion of omics technologies, the challenges remain the same: translating new discoveries into tangible solutions for animal agriculture and biomedical science. The vast increase in data from -omics has illuminated new biological pathways and mechanisms; however, the practical integration and application of these findings into meat production, animal health, and disease prevention has lagged. Modern molecular biology tools have provided us unprecedented access to the vast and intricate networks regulating muscle growth and function; grabbing this opportunity to integrate the hypothesis-generating -omics data with traditional, hypothesis-driven research lies in the potential to address economically costly myopathies, such as Wooden Breast disease in poultry.

In conclusion, integration of a traditional hypothesis-driven research with modern -omics approaches represent a unique and powerful opportunity to close the gap that has persisted for decades. By embracing both strategies, we can advance our understanding of muscle biology and deliver the practical benefits envisioned over 50 years ago. Therefore, the challenges are clear: ensuring that the wealth of data generated by -omics technologies does not remain disconnected from practical applications but instead leads to developing innovative and actionable solutions that improve animal health, meat quality, and beyond.
